# Meditative-based diaphragmatic breathing vs. vagus nerve stimulation in the treatment of fibromyalgia—A randomized controlled trial: Body vs. machine

**DOI:** 10.3389/fneur.2022.1030927

**Published:** 2022-11-03

**Authors:** Charles Ethan Paccione, Audun Stubhaug, Lien My Diep, Leiv Arne Rosseland, Henrik Børsting Jacobsen

**Affiliations:** ^1^Division of Emergencies and Critical Care, Department of Pain Management and Research, Oslo University Hospital, Oslo, Norway; ^2^Faculty of Medicine, Institute of Clinical Medicine, University of Oslo, Oslo, Norway; ^3^Mind-Body Lab, Department of Psychology, University of Oslo, Oslo, Norway; ^4^Oslo Center for Biostatistics and Epidemiology, Oslo University Hospital, Oslo, Norway; ^5^Division of Emergencies and Critical Care, Department of Research and Development, Oslo University Hospital, Oslo, Norway

**Keywords:** meditation, diaphragmatic breathing, vagus nerve, vagus nerve stimulation (VNS) therapy, chronic pain and fibromyalgia, heart rate variability (HRV), pain intensity

## Abstract

**Importance:**

Vagus nerve innervation *via* electrical stimulation and meditative-based diaphragmatic breathing may be promising treatment avenues for fibromyalgia.

**Objective:**

Explore and compare the treatment effectiveness of active and sham transcutaneous vagus nerve stimulation (tVNS) and meditative-based diaphragmatic breathing (MDB) for fibromyalgia.

**Design:**

Participants enrolled from March 2019–October 2020 and randomly assigned to active tVNS (*n* = 28), sham tVNS (*n* = 29), active MDB (*n* = 29), or sham MDB (*n* = 30). Treatments were self-delivered at home for 15 min/morning and 15 min/evening for 14 days. Follow-up was at 2 weeks.

**Setting:**

Outpatient pain clinic in Oslo, Norway.

**Participants:**

116 adults aged 18–65 years with severe fibromyalgia were consecutively enrolled and randomized. 86 participants (74%) had an 80% treatment adherence and 107 (92%) completed the study at 2 weeks; 1 participant dropped out due to adverse effects from active tVNS.

**Interventions:**

Active tVNS is placed on the cymba conchae of the left ear; sham tVNS is placed on the left earlobe. Active MDB trains users in nondirective meditation with deep breathing; sham MDB trains users in open-awareness meditation with paced breathing.

**Main outcomes and measures:**

Primary outcome was change from baseline in ultra short-term photoplethysmography-measured cardiac-vagal heart rate variability at 2 weeks. Prior to trial launch, we hypothesized that (1) those randomized to active MDB or active tVNS would display greater increases in heart rate variability compared to those randomized to sham MDB or sham tVNS after 2-weeks; (2) a change in heart rate variability would be correlated with a change in self-reported average pain intensity; and (3) active treatments would outperform sham treatments on all pain-related secondary outcome measures.

**Results:**

No significant across-group changes in heart rate variability were found. Furthermore, no significant correlations were found between changes in heart rate variability and average pain intensity during treatment. Significant across group differences were found for overall FM severity yet were not found for average pain intensity.

**Conclusions and relevance:**

These findings suggest that changes in cardiac-vagal heart rate variability when recorded with ultra short-term photoplethysmography in those with fibromyalgia may not be associated with treatment-specific changes in pain intensity. Further research should be conducted to evaluate potential changes in long-term cardiac-vagal heart rate variability in response to noninvasive vagus nerve innervation in those with fibromyalgia.

**Clinical trial registration:**

https://clinicaltrials.gov/ct2/show/NCT03180554, Identifier: NCT03180554.

## Introduction

Fibromyalgia (FM) is a chronic disorder characterized by disabling pain that lasts for 3 months or more in multiple regions of the body and is commonly associated with psychological distress (1). Global prevalence rates for FM are estimated to be 4.9 % in women and 2.9 % in men (2, 3) with a treatment cost of $12-14 billion each year in the United States alone^1^. Currently available FM treatments provide only modest improvements in pain and minimum improvements in psychophysiological functioning (4–11). The complex etiology of FM makes it continually difficult to develop targeted treatment strategies that are effective, affordable, and safe.

The presence of autonomic nervous system (ANS) dysfunction in those with FM (12, 13) is significantly associated with high self-report pain intensity when compared to pain-free controls (14). A frequently reported indication of ANS dysfunction in FM is low resting-state heart rate variability (HRV) (15). HRV represents the change in the time interval between successive heartbeats and is one of the most important indexes of cardiovascular and autonomic health in clinical and nonclinical populations (16–22). Compared to healthy controls, those diagnosed with FM display significantly lower levels of cardiac vagal tone— specific HRV variables thought to indirectly measure activity of the vagus nerve, the main nerve of the parasympathetic nervous system (19, 23–26).

Vagus nerve stimulation (VNS), which typically involves electrical stimulation of the vagal nerve, is an approved therapy for both refractory epilepsy and treatment-resistant depression (26, 27). Preliminary intervention trials on humans have shown that electrical VNS reduces widespread pain in patients with treatment-resistant FM (28) and reduces sensitivity of mechanically evoked pain in healthy volunteers (29). In addition to electrical-based VNS, cardiorespiratory VNS in the form of meditative-based diaphragmatic breathing (MDB) can also be used to directly influence brain electrical activity mediated by the vagus nerve arising from the diaphragm (30). MDB techniques demonstrate positive analgesic effects for some acute pain conditions but have yet to be determined effective for those with FM (7). These findings suggest that an indirect mediation of pain intensity through changes in cardiac-vagal tone may be a pathway to alleviate pain and disability in FM (31). To date, experimental evidence elucidating the underlying psychophysiological mechanisms is lacking.

The primary aim of this single-blinded randomized sham-controlled clinical trial was to evaluate and compare the effects of two noninvasive and portable means of electrical- and cardiorespiratory-based VNS—transcutaneous vagus nerve stimulation (tVNS) and meditative-based diaphragmatic breathing (MDB)—on photoplethysmography (PPG) measured cardiac vagal HRV and average self-report numeric rating scale (NRS) pain intensity. We hypothesized that adults with FM randomized to receive active MDB or active tVNS would display greater increases in HRV as compared to those randomized to sham MDB or sham tVNS after 2-weeks of treatment. Furthermore, we hypothesized that a change in HRV would be correlated with a change in self-reported NRS pain intensity. Lastly, we hypothesized that active treatments would outperform sham treatments on all clinical pain-related secondary outcomes.

## Materials and methods

### Setting and procedure

**Figure 1:**
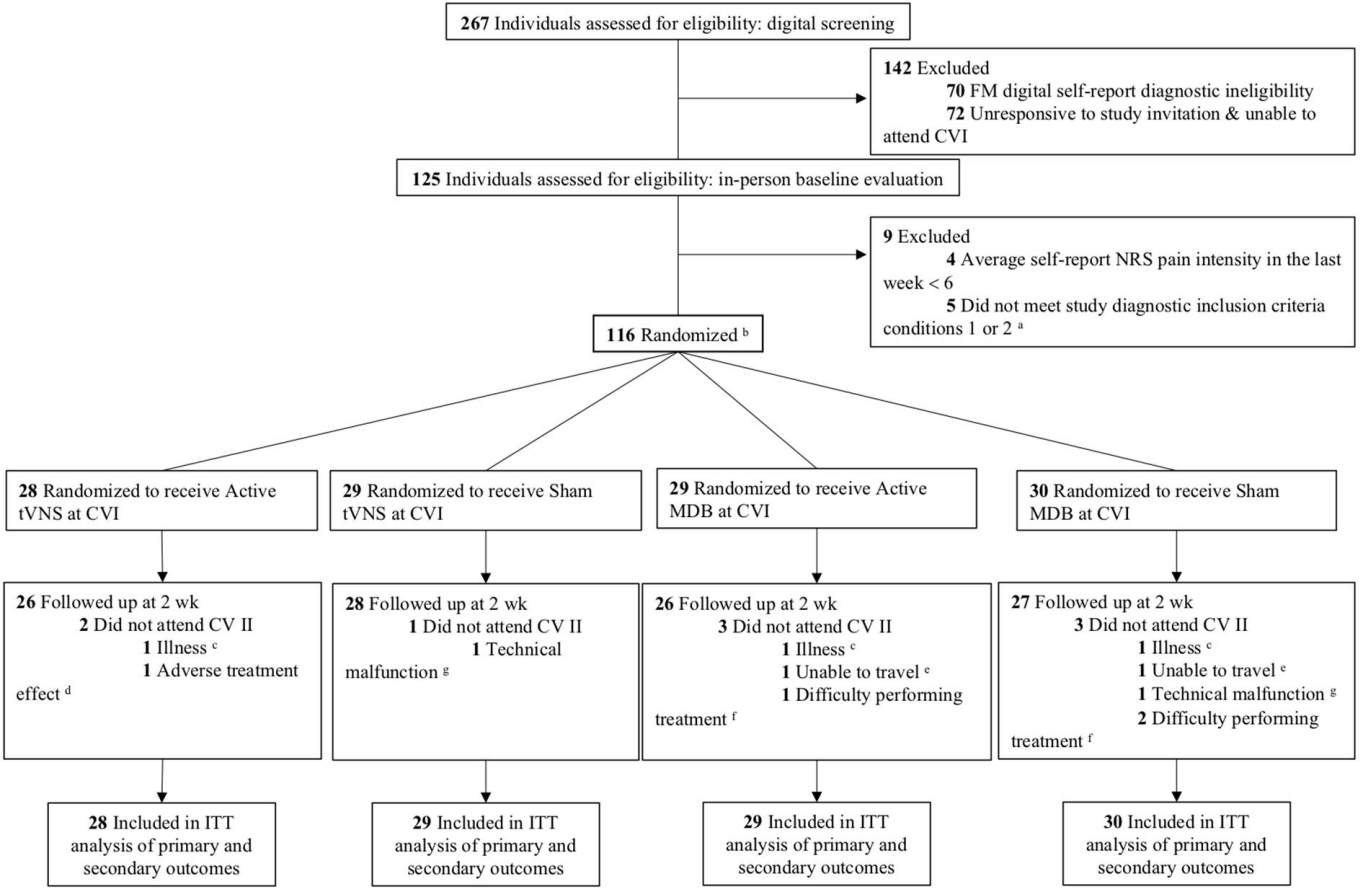
CONSORT flow diagram of study participation. ^**a**^Condition 1: Widespread pain index (WPI) ≥ 7 and symptom severity scale (SSS) score ≥ 5; Condition 2: WPI of 4–6 and SSS score ≥ 9. ^**b**^Of the 116 participants randomized, 86 completed 80% of their assigned home treatment (i.e., 23 out of 28 total treatment sessions) which was regarded as adequate treatment adherence; tVNS version 1: 24 participants compliant; tVNS version 2: 25 participants compliant; MDB version 1, 20 participants compliant; MDB version 2, 17 participants compliant. ^**c**^Illness: tVNS version 1, 1 participant discontinued treatment due to a diagnosis of a comorbid condition; MDB version 1, 1 participant discontinued treatment due to sickness; MDB version 2, discontinued treatment due to excessive fatigue. ^**d**^Adverse treatment effects: tVNS version 1, 1 participant discontinued treatment due to chest discomfort and additional pain due to tVNS stimulation. ^**e**^Unable to travel: MDB version 1, 1 participant discontinued treatment due to unknown circumstances and trouble traveling to the clinic; MDB version 2, 1 participant discontinued treatment due to trouble traveling to the clinic. ^**f**^Difficulty performing treatment: MDB version 1, 1 participant discontinued treatment due to difficulty with following the breathing pattern; MDB version 2, 1 participant discontinued treatment due to difficulty with the meditation posture. ^**g**^Technical malfunction: tVNS version 2, 1 participant discontinued treatment due to technical issues with their ear electrode; MDB version 2, 1 participant discontinued treatment due to technical issues with the MDB intervention app and HRV app. CVI, Clinical Visitation I; CVII, Clinical Visitation II; tVNS, transcutaneous vagus nerve stimulation; MDB, Meditative-based diaphragmatic breathing; ITT, intent-to-treat.

Study design and clinical trial protocol was peer-reviewed and published [see trial protocol (31)]. FM participants were consecutively recruited from, (1) the Department of Pain Management and Research at Oslo University Hospital, in Oslo, Norway; (2) the surrounding Oslo community; and (3) from the south-east region of Norway. Information describing the trial was posted on ClinicalTrials.gov (Identifier: NCT03180554) and CRISITIN (Current Research Information System in Norway). Interested participants were directed to the Oslo University Hospital website where they logged into a secure digital data collection system and filled out a brief self-report inclusion/exclusion form. Participants who met self-reported inclusion criteria received an appointment for both clinical visitation I (CVI) and clinical visitation II (CVII) at the Department of Pain Management and Research, Division of Emergencies and Critical Care, Oslo University Hospital (Figure 1). Inclusion and exclusion criteria were assessed using data from the self-report digital inclusion/exclusion form and an in-person diagnostic screening interview at CVI. Participants enrolled between March 2019 and October 2020. Ethical approval was obtained by the Regional Committee for Medical and Health Research Ethics (REC South-East, Project Number: 2017/7066) in Norway in May 2017.

### Participants and blinding

Participants were 18–65 years of age with severe FM that persisted at least 3 months or more with an average self-report NRS pain intensity of 6-10. A full list of study exclusion criteria can be found in trial protocol (31). Participants provided both oral and written informed consent for participation. Active and sham treatment allocation was concealed from the participants and testing administrators—both were told that they will provide/receive two different versions of nerve stimulation at different locations on the ear (for the tVNS group) or that there are two breathing techniques that are being explored (for the MDB group). The testing administrators introduced either “Version 1” (active) or “Version 2” (sham) of the respective treatment interventions. The principal investigators were blinded to patient treatment allocation as well as the randomization.

### Randomization

Participants were consecutively randomized to one of the four treatment groups which were ran in parallel: tVNS #1 (active); tVNS #2 (sham); MDB #1 (active); MDB #2 (sham). The computer-generated randomized allocation sequencer was imported into an electronic data capture system and made available to testing administrators. The randomization was stratified by sex (male = 1, female = 2) and cardioactive medications (yes = 1, no = 0) with varying block size within strata. Participants were permitted to continue taking any previously prescribed pain medications during the trial period.

### Interventions

Both treatment types (MDB or tVNS) and versions (active or sham) were self-delivered for 15 min in the morning upon waking and 15 min at night before bed for a total duration of 2-weeks [see intervention details in (31)]. Participants received a step-by-step training session and instruction manual on how to self-deliver their assigned treatment at home for the 14-day period at Clinical Visitation I (CVI). Treatment adherence was evaluated at the end of CVII by testing administrators by a combination of HRV recording sessions and respiratory data (for active and sham MDB).

Active and sham MDB was delivered *via* an android smartphone-app program (32) and compatible CE-approved respiratory gating device (31). Active MDB guided participants in nondirective meditation (33) and slow diaphragmatic breathing (6 breadths/min) (17) whereas sham MDB instructed participants in focused paced breathing (22, 34, 35) at 12 breadths/min—the normal resting respiration rate for an adult (36). Active and sham auricular transcutaneous vagus nerve stimulation (tVNS) was delivered utilizing two titanium electrodes connected to a portable electrical stimulator (22, 37–39). Participants placed the bipolar stimulation electrode within the conchae of the left ear (active tVNS) or on the center of the left earlobe (40, 41) (sham tVNS). A detailed description of tVNS and MDB delivery is described in (31).

### Follow-up

Testing administrators collected data in-person at baseline (CVI) and post-intervention follow-up (CVII).

### Measures

FM diagnostic information and cardiovascular information was obtained at baseline (Table 1). Primary and secondary outcome measures were collected at baseline and post-intervention follow-up.

**Table 1:** Baseline characteristics of participants by treatment group.

**Baseline characteristics**	**Mean (SD)**				
	**Total** **(*N* = 116)**	**Active tVNS** **(*n* = 28)**	**Sham tVNS** **(*n* = 29)**	**Active MDB** **(*n* = 29)**	**Sham MDB** **(*n* = 30)**
Female, N (%)	110 (94.8)	27 (96.4)	28 (96.6)	27 (93.1)	28 (93.3)
Age (yrs)	45.69 (10.25)	48.25 (8.88)	45.45 (12.04)	46.76 (10.43)	42.50 (8.95)
Body mass index (BMI) (kg/m^2^)	29.33 (6.35)	29.26 (6.49)	29.37 (5.49)	29.57 (7.90)	29.11 (5.59)
Waist-to-hip ratio (WHR)	0.83 (0.08)	0.83 (0.07)	0.83 (0.10)	0.82 (0.08)	0.85 (0.07)
Systolic blood pressure (SBP) (mm Hg)	119.24 (14.21)	117.82 (15.27)	121.54 (14.48)	115.10 (13.77)	122.43 (12.82)
Diastolic blood pressure (DBP) (mm Hg)	77.76 (9.09)	77.57 (9.77)	79.07 (8.92)	76.14 (9.31)	78.27 (8.56)
Average resting heart rate (HR) (BPM)	62.69 (9.60)	58.88 (9.58)	63.12 (9.15)	64.93 (10.43)	63.68 (8.61)
Average interval between heart beats (AVNN) (ms)	983.50 (155.55)	1049.42 (163.16)	971.11 (145.94)	950.37 (166.52)	968.17 (135.56)
***N*** **(%)**					
Spouse/Partner?	75 (65.8)	21 (75.0)	20 (71.4)	17 (60.7)	17 (56.67)
Daily caffeinated coffee and/or tea consumption (cups/day), mean (SD)	2.85 (2.04)	3.0 (1.59)	2.71 (1.98)	2.93 (1.29)	2.77 (2.33)
Smoke currently, yes	12 (10.5)	2 (7.1)	3 (10.7)	3 (10.7)	6 (20.0)
Alcohol consumption 4 or more days/week, yes	10 (8.8)	3 (10.7)	0	5 (17.9)	2 (6.7)
Exercise about every day, yes	34 (29.82)	4 (19.3)	11 (39.3)	12 (42.9)	7 (23.3)
Snuff or chewing tobacco daily consumption, yes	5 (4.4)	0	0	2 (7.1)	3 (10.0)
Antidepressants	23 (19.8)	3 (10.7)	7 (24.1)	6 (20.7)	7 (23.3)
Cardioactive medication currently taking, yes	87 (75.0)	20 (71.4)	22 (75.9)	22 (75.9)	23 (76.7)
Blood pressure medication currently taking, yes	21 (18.10)	6 (21.43)	4 (13.79)	6 (20.69)	5 (16.67)
Cholesterol medication currently taking, yes	9 (7.76)	4 (14.29)	2 (6.90)	2 (6.90)	1 (3.33)
Anxiolytics currently taking, yes	6 (5.17)	1 (3.57)	1 (3.45)	1 (3.45)	3 (10.00)
Prescription pain medication (analgesics) currently taking, yes	40 (34.48)	10 (35.71)	8 (27.59)	12 (41.38)	10 (33.33)
Non- prescription pain medication (non-analgesic) currently taking, yes	14 (12.1)	4 (14.3)	4 (13.8)	2 (6.9)	4 (13.3)
Sleeping pills currently taking, yes	23 (19.8)	5 (17.86)	8 (27.59)	8 (27.59)	2 (6.67)
Hormonal contraceptives (women only), yes	36 (32.7)	3 (11.1)	10 (35.7)	10 (37.0)	13 (46.4)
Menopause hormones taking (women only), yes	13 (12.0)	2 (7.7)	4 (14.8)	3 (11.1)	4 (14.3)
**Baseline measures of primary HRV outcome values, median (IQR)**					
rMSSD (ms)	42.25 (32.25–59.65)	48.2 (39.1–57.6)	39.0 (31.8–59.5)	39.2 (29.8–50.6)	41.7 (27.6–66.9)
hfHRV (ms^2^)	0.046 (0.033–0.068)	0.054 (0.04–0.07)	0.05 (0.03–0.06)	0.05 (0.04–0.07)	0.04 (0.03–0.09)
pNN50, mean (SD) (%)	25.41 (18.92)	27.99 (14.58)	24.06 (19.61)	23.07 (18.60)	26.65 (22.27)
**Baseline measures of secondary outcome scores, mean (SD)**					
**Clinical pain outcomes**					
Numerically rated scale (NRS) current pain intensity (0–10), mean (SD)	5.49 (1.77)	5.57 (1.55)	5.62 (1.82)	5.69 (2.16)	5.10 (1.52)
FM widespread pain index (WPI) (0–19), mean (SD)	14.26 (2.67)	14.82 (2.65)	14.38 (3.14)	13.79 (2.38)	14.07 (2.46)
FM symptom severity scale (SSS) (0–12), mean (SD)	9.19 (1.87)	9.64 (1.91)	8.97 (2.03)	8.97 (1.94)	9.20 (1.61)
FM severity (FS) scale (WPI + SSS) (0–31), mean (SD)	23.45 (3.68)	24.45 (3.42)	23.34 (4.45)	22.76 (3.37)	23.27 (3.34)
Average numerically rated scale (NRS) pain intensity in the last week (0–10), mean (SD)	6.72 (0.74)	6.68 (0.77)	6.83 (0.80)	6.79 (0.73)	6.60 (0.77)
**Treatment credibility and expectancy, mean (SD)**					
At this point, how logical does the treatment offered to you seem? (1-9)	6.57 (1.81)	6.39 (1.87)	6.64 (1.68)	6.54 (1.55)	6.70 (2.15)
At this point, how successfully do you think this treatment will be in raising the quality of your functioning? (1-9)	5.50 (1.66)	5.71 (1.90)	5.68 (1.16)	5.04 (1.32)	5.57 (2.06)
How confident would you be in recommending this treatment to a friend who experiences similar problems? (1-9)	5.39 (2.23)	5.86 (2.19)	5.11 (1.93)	5.54 (2.03)	5.10 (2.68)
By the end of the treatment, how much improvement in your function do you think will occur? (0-100%)^*^	39.91 (19.39)	43.93 (21.49)	45.36 (17.10)	31.07 (17.07)	39.33 (19.29)
At this point, how much do you really feel that the treatment will help you to improve your functioning? (1-9)**^∧^**	5.18 (1.80)	5.86 (1.72)	5.36 (1.62)	4.54 (1.60)	5.0 (2.03)
By the end of the treatment, how much improvement in your functioning do you really feel will occur? (0-100%)	37.72 (22.34)	43.21 (23.10)	38.93 (21.66)	30.36 (18.75)	38.33 (24.51)
Credibility Score	5.82 (1.61)	5.99 (1.67)	5.81 (1.22)	5.70 (1.32)	5.79 (2.12)
Expectancy Score	27.61 (13.69)	31 (14.71)	29.88 (12.41)	21.99 (11.81)	27.56 (14.45)

* p = 0.024 for differences between groups.

∧ p = 0.041 for differences between groups.

### Primary outcome

Resting-state photoplethysmography (PPG)-measured HRV data was obtained *via* an Android App utilized in prior research trials (42–44) and validated with the Polar H7 device and electrocardiography (ECG) (45) at CVI and CVII. HRV recordings of 1 min were used to assess cardiac-vagal HRV parameters: the root mean square of successive differences (rMSSD), high-frequency (HF) HRV, and the percentage of successive RR intervals that differ by more than 50 ms (pNN50). PPG-measured HRV is considered a reliable means of computing HRV if the correct procedural methods of recording are implemented in trials (46): each participant was instructed to remain seated for at least 5 min prior to taking the first HRV measurement to acclimatize; acclimatization reduces HRV changes due to posture changes (47, 48) and help reduce confounds driven by participant test anxiety and changes in respiration (18, 49, 50). Immediately following the 5 min acclimatization period, three 1- min HRV recordings were taken on the tip of the right index finger of each participant separated by 1-min intervals [recording procedure is provided in (31)]. Due to the impact of attentive states and test anxiety on respiratory frequency and HRV recordings (51), the beginning and end of each of the three HRV recordings were not announced. An average of the last two recordings was used as the baseline measure.

### Secondary outcomes

Testing administrators conducted a FM clinical interview at CVI and in CVII. Participants were guided through an electronic version of the 2016 revision to the 2010/2011 FM diagnostic criteria form (52) which computes an overall FM severity (0–31 point scale, where higher numbers indicated more severe pain status) composed of a widespread pain index (WPI) score and a symptom severity scale (SSS) score. The numeric rating scale (NRS) (53) was used to assess average pain intensity in the last week at both CVI and CVII. Participants chose a number between 0 and 10 that best described their pain intensity—zero indicates “no pain at all” whereas the upper limit represents “the worst pain ever possible.”

Participant-reported outcome measures in the form of questionnaires were completed electronically by each participant at the end of CVI and CVII [for a complete list of questionnaires see (31)]. The Credibility/Expectancy Questionnaire (CEQ) was delivered at CVI and measures a participant's expectations about the efficacy of a particular treatment and whether they think that the treatment is credible or not; it is composed of six items which are scored on a 9-point scale ranging from “not at all logical,” “somewhat logical,” and “very logical.” Items 4 and 6 ask the participant how they feel and how they think the administered treatment will improve their overall health state regarding their pain on a 0–100% scale, where 0% represents “no improvement” whereas 100% represents “total improvement.”

### Adverse events

Adverse events were identified during intervention period and by follow-up testing administrator questions about significant discomfort, technical usability issues, or harm of any kind caused by the interventions.

### Sample size

A sample size of at least 84 participants (21 in each group) was needed to detect meaningful differences between our four groups at 2 weeks (54, 55). Effect size distributions of 0.25, 0.5, and 0.9 were interpreted as small, medium, and large effects (after rounding to the closest 0.05). To account for possible participant dropout during the trial, we aimed to recruit a minimum of 112 participants (28 participants per group) which would correlate to a statistical power of 0.9. A final population of *N* =116 were formally enrolled and randomized (Figure 1) for the study. This recommended sample size is based upon the effect size distributions and was tailored to our specific study to appropriately power the investigation and make it more likely to better replicate and derive true effect size estimates for both HRV and NRS pain intensity (55).

### Statistical analyses

The analyses were performed with STATA/SE for Windows, Version 16.1 and IBM SPSS Statistics version 27 (56, 57). Characteristics of the four groups were described by means and standard deviations or medians and quartile ranges for continuous variables. Frequencies and percentages were given for categorical variables/data. Missing data after baseline were handled with last observation carried forward method as specified in the protocol. Associations between changes in HRV and pain were examined by correlation analyses—strengths of the intercorrelations were assessed by Pearson's and Spearman's correlation coefficients. A linear mixed model for continuous outcomes was used to fit two data points (week 0, week 2) per patient to include all patients in the analysis in accordance with the Intention to Treat principle (ITT).

Difference in change from baseline between the four groups with and without adjusting for baseline was tested by using two linear mixed effect models for continuous outcome data and a likelihood ratio test. For the random part of the linear mixed effect model, participant identifier or identifier and time, were specified as the random variables. In the linear mixed model with two random variables (identifier and time), an exchangeable variance-covariance structure was specified. For the fixed part of the mixed model, group and time and interaction between group and time were specified to test whether the change differed between the groups over time with and without adjusting for baseline values. Highly skewed (non-normally distributed) variables were logarithmically transformed before fitting the mixed models. The analyses of average changes between groups over time were carried out for the compliant groups (*n*= 86) and full sample (*N*= 116).

## Results

Among 267 individuals expressing interest in study participation and screened for eligibility, 116 were enrolled and randomized (Figure 1). Of the 116 participants randomized, 86 (74%) in total completed 80% of their assigned home treatment (i.e., 23 out of 28 total treatment sessions) which was regarded as adequate treatment adherence (see Figure 1 for 80% compliancy numbers by treatment group) and 107 (92%) were seen at CVII for post-treatment follow-up.

Treatment groups were similar in baseline characteristics and current cardioactive medication use. Significant between-group differences were found for hormonal contraceptive use among women and two items on the CEQ regarding improvement in function (Table 1). The mean (SD) pain intensity in the past week at baseline was 6.72 (0.74) for the total population indicating severe intensity. No significant differences were found across treatment groups at baseline for any cardiac vagal HRV variables. A pairwise Spearman correlation analysis (Table 2) performed for the entire FM population (*N* = 116) which explored associative changes from CVI to CVII among HRV and pain variables showed that NRS average pain intensity in the last week had no significant correlations with changes in HRV.

**Table 2:** Pairwise correlations of change from clinical visitation I to clinical visitation II between HRV, FM pain variables, and psychological distress (*N* = 116).

**Heart rate** **variability** **(HRV)**	**NRS average** **pain intensity** **(0–10)**	***P*** **-value**	**NRS current** **pain intensity** **(0–10)**	***P*** **-value**	**Widespread** **pain** **index (WPI)** **(0–19)**	***P*** **-value**	**Symptom** **severity** **scale (SSS)** **(0–12)**	***P*** **-value**	**Fibromyalgia** **severity (FS)** **(0–31)**	***P*** **-value**
rMSSD (ms)	−0.07	0.444	0.09	0.315	−0.06	0.515	0.06	0.537	0.02	0.867
hfHRV (ms^2^)	−0.172	0.066	−0.01	0.884	−0.02	0.826	0.12	0.204	0.03	0.726
pNN50 (%)	−0.04	0.682	0.03	0.777	0.06	0.528	0.08	0.379	0.09	0.341

Spearman correlation coefficients are given for rMSSD and hfHRV because they were not normally distributed. Pearson correlation coefficients were given for pNN50.

### Primary outcome

There were no significant differences found across treatment groups in any of the cardiac-vagal variables of interest (Table 3A).

**Table 3:** Primary outcome: Mean (95% CI) differences between treatment groups at 2 wk follow-up for HRV and total average change in HRV from baseline to follow-up for total study population (A; *N* = 116) and treatment compliancy population (B; *n* = 86) (Imputed analyses adjusted for baseline differences).

	**A) Total study population (*****N*** = **116)**		**Active tVNS (*****n*** = **28)**		**Sham tVNS (*****n*** = **29)**		**Active MDB (*****n*** = **29)**		**Sham MDB (*****n*** = **30)**		
**Heart rate** **variability** **(HRV)**	**Mean change** **(95% CI)**	**Overall *P*-** **value for** **difference** **between CVI and** **CVII**	**Mean change** **(95% CI)**	***p*** **- value**	**Mean change** **(95% CI)**	***p*** **- value**	**Mean change** **(95% CI)**	***p*** **- value**	**Mean change** **(95% CI)**	***p*** **- value**	**Overall *P*-** **value for** **difference between** **groups at** **CVII**
rMSSD^*^ (ms)	0.95 (0.87, 1.03)	0.186	1.00 (0.90, 1.13)	0.942	0.93 (0.84,1.05)	0.248	0.96 (0.86, 1.08)	0.518	1.03 (0.92, 1.14)	0.654	0.672
hfHRV* (ms^2^)	0.95 (0.87, 1.05)	0.311	0.83 (0.68, 1.00)	0.050	1.02 (0.84, 1.23)	0.841	0.97 (0.81, 1.17)	0.778	0.99 (0.83, 1.19)	0.924	0.411
pNN50 (%)	−0.93 (−3.12, 1.26)	0.403	−0.72 (−4.87, 3.43)	0.734	−1.07 (−5.07, 2.93)	0.601	−0.64 (−4.71, 3.44)	0.758	0.99 (−2.95, 4.92)	0.623	0.893
	**B) Treatment compliancy population (*****n*** = **86)**		**Active tVNS (*****n*** = **24)**		**Sham tVNS (*****n*** = **25)**		**Active MDB (*****n*** = **20)**		**Sham MDB (*****n*** = **17)**		
**Heart rate** **variability** **(HRV)**	**Mean change** **(95% CI)**	**Overall** *****P***-value for** **difference** **between** **CVI and** **CVII**	**Mean change** **(95% CI)**	***p*** **-value**	**Mean change** **(95% CI)**	***p*** **-value**	**Mean change** **(95% CI)**	***p*** **-value**	**Mean change** **(95% CI)**	***p*** **-value**	**Overall** *****P***-value for** **difference between** **groups at** **CVII**
rMSSD* (ms)	0.93 (0.84, 1.04)	0.221	1.04 (0.86, 1.27)	0.673	0.92 (0.77, 1.12)	0.414	0.88 (0.71, 1.07)	0.209	1.03 (0.83, 1.30)	0.761	0.569
hfHRV* (ms^2^)	0.98 (0.88, 1.10)	0.774	0.81 (0.66, 0.99)	0.043	1.09 (0.90, 1.32)	0.378	1.01 (0.81, 1.25)	0.942	1.06 (0.84, 1.34)	0.602	0.174
pNN50 (%)	−1.25 (-4.12, 1.61)	0.391	−0.06 (-5.56, 5.44)	0.984	−1.89 (-7.17, 3.38)	0.482	−3.18 (-9.08, 2.72)	0.291	0.33 (-6.07, 6.73)	0.920	0.830

* The HRV variables were transformed from original to logarithmic scale before fitting the linear mixed model. The statistical results from the mixed model were transformed back to the original scale and reported in the table. Three observations of hfHRV had null values which were excluded before using log-transformation.

### Per-protocol analyses

No significant across-group differences were found in any of the cardiac-vagal variables of interest for participants who achieved 80% treatment compliancy (a completion of 23 out of 28 total treatment sessions) (*n* = 86) (Table 3B).

### Secondary outcomes

Significant across group differences were found for current pain intensity with significant within-group changes in those randomized to active tVNS (−0.82; 95% CI, −1.32−0.31) and sham tVNS (−0.86; 95% CI, −1.36–−0.36) (Table 4). Significant across group differences were also found for overall FM severity. No significant across group differences were found for FM symptom severity, widespread pain, or average pain intensity in the last week.

**Table 4:** Mean (95% CI) differences between treatment groups at 2 wk follow-up and total average change from baseline to follow-up for secondary outcomes (Imputed analyses adjusted for baseline differences; *N* = 116).

**Secondary** **outcome** **measures**	**Total (*****N*** = **116)**		**Active tVNS (*****n*** = **28)**		**Sham tVNS (*****n*** = **29)**		**Active MDB (*****n*** = **29)**		**Sham MDB (*****n*** = **30)**		
	**Mean** **(95% CI)**	**Overall** ***P*-value** **for** **difference** **between CVI and** **CVII**	**Mean** **(95% CI)**	***p*** **-value**	**Mean** **(95% CI)**	***p*** **-value**	**Mean** **(95% CI)**	***p*** **-value**	**Mean** **(95% CI)**	***p*** **-value**	**Overall** ***P*-value** **for** **difference** **between** **groups** **at CVII**
Overall FM severity (FS) (0–31)	−2.08 (−2.58, −1.58)	<0.001	−2.82 (−3.83, −1.81)	<0.001	−2.90 (−3.89, −1.91)	<0.001	−1.28 (−2.27, −0.28)	0.012	−1.37 (−2.34, −0.39)	0.006	0.025
NRS average pain intensity in the last week (0–10)	−0.59 (−0.71, −0.46)	<0.001	−0.57 (−0.83, −0.31)	<0.001	−0.86 (−1.11, −0.61)	<0.001	−0.59 (−0.84, −0.33)	0.001	−0.33 (−0.58, −0.08)	0.009	0.199
NRS current pain intensity (0–10)	−0.38 (−0.63, −0.13)	0.003	−0.82 (−1.32, −0.31)	0.002	−0.86 (−1.36, −0.36)	0.001	−0.07 (−0.57, 0.43)	0.787	0.21 (−0.29, 0.69)	0.425	0.004
Widespread pain index (WPI) (0–19)	−1.15 (−1.50, −0.79)	<0.001	−1.50 (−2.23, −0.77)	<0.001	−1.69 (−2.39, −0.98)	<0.001	−0.79 (−1.51, −0.08)	0.029	−0.63 (−1.33, 0.07)	0.077	0.098
Symptom severity scale (SSS) (0–12)	−0.93 (−1.22, −0.65)	<0.001	−1.32 (−1.91, −0.74)	<0.001	−1.21 (−1.78, −0.63)	<0.001	−0.48 (−1.06, 0.09)	0.098	−0.73 (−1.31, −0.17)	0.011	0.134

### Adverse events

One participant randomized to receive active tVNS experienced chest discomfort and additional pain due to the tVNS stimulation and decided to discontinue treatment. No other serious adverse events were reported in this trial.

## Discussion

The aim of our randomized controlled clinical trial was to establish and compare the efficacy of active and sham versions of tVNS and MDB on cardiac vagal tone and average pain intensity in adults diagnosed with severe FM. Contrary to our primary hypothesis, no significant across-group differences in cardiac vagal tone were found. Furthermore, contrary to our second hypothesis, no significant associations were found between changes in cardiac-vagal HRV and changes in average NRS pain intensity during treatment in our entire FM population; active treatments did not outperform sham treatments for clinical pain-related secondary outcomes. However, significant group differences were found for overall FM severity and current pain intensity at post-treatment follow-up.

The presence of significant pain reductions in the absence of significant across-group differences in cardiac-vagal HRV variables may challenge the current notion that rMSSD and/or hfHRV are reliable indexes of cardiac vagal activity (19, 24, 25, 58). Recent preliminary findings in rats (59) and in healthy subjects (60) indicate that there is no correlation between cardiac vagal HRV and tonic vagal activity or vagal stimulation over time (61). These findings may suggest three important implications: (1) that clinical measures of HRV may not represent vagal activity; (2) the term “vagal tone” may be misleading; and/or (3) rMSSD may only reflect a small subset of cardiac vagal *afferent* activity.

Utilizing ultra short-term (<5 min) PPG methods for evaluating cardiac-vagal HRV could have aided in increasing participant treatment compliancy and regimen (62) while also decreasing overall clinical visitation time and associated costs (63). However, it may have been insufficient in detecting potentially significant autonomic changes in participants before, during, and after treatment. Long-term HRV recordings still represent the typical reference standard for predicting health outcomes whereas short-term values are proxies of long-term values with unknown predictive validity; therefore, ultra-short HRV measurements could be considered as “proxies of proxies” (63). Despite the fact that our HRV recording method, instrumentation, and procedure has been validated with both the Polar H7 device and electrocardiography (ECG) (42–45) evaluating HRV utilizing classic 5-min ECG recording windows during pre and post clinical visitations could have aided in determining whether the observed changes in pain intensity were associated with changes in HRV (64). Based on clinical theories such as the vagal-tank theory, longer vagus nerve innervation treatment methods for those with FM may be needed to detect a significant perturbance of low HRV levels and help understand how the ≪vagal tank≫ sustains self-regulatory efforts to build a higher resting cardiac vagal control over time, yet this is speculative (65).

Significant changes in overall FM severity were found in all groups. Only those who received active or sham tVNS displayed significant changes in current NRS pain intensity as compared to those randomized to active or sham MDB, yet the clinical significance of these findings are questionable: overall FM severity is based on a 0–31 point scale (52) whereas current pain intensity is known to (1) drastically fluctuate daily in those with FM; (2) is mediated by psychological distress in relation to physical functioning; and (3) is therefore not considered a reliable direct indicator of treatment efficacy (66). Furthermore, the overall effects of active and sham versions of tVNS in our trial were quite similar.

Observational fMRI analyses (40, 67) showed that active tVNS stimulation at the cymba conchae and sham tVNS stimulation of the earlobe resulted in similar overlapping BOLD changes in cortical areas. These observational results may provide support for our clinical findings which showed an overall nonsignificant difference of clinical efficacy between active and sham tVNS indicating that the terms “active” and “sham” tVNS stimulation could be somewhat misleading (68). More pronounced effects in favor of active respiratory biofeedback as compared to sham (34, 36, 69, 70) have been found in previous clinical trials (71–76). However, similar effects for changes in overall FM severity and average NRS self-report pain intensity for active and sham MDB in this trial challenges the significant differentiable effects demonstrated in prior literature.

### Strengths and limitations

There are several important limitations to consider. Firstly, sociodemographic characteristics including education, annual income, and employment were not collected in this trial and could have provided pertinent information for describing our findings. Secondly, even though this study was practiced as a double-blinded clinical trial, it was formally categorized as a single-blind trial because there was no way of ensuring that either the testing administrators or the participants held any prior insights into active and inactive MDB/tVNS treatment protocols. Furthermore, to sufficiently detect a difference between groups in HRV as it relates to NRS pain intensity, a sample size between 30 and 77 (depending on the HRV metric of interest) is typically recommended (77); subgroups are commonly employed within designs that have been suggested to require 20 participants per cell (54). However, due to the exploratory nature of this investigation, our sample size determination may not have been ideal to detect clinically significant differences between our groups. Lastly, it is important to note our choice of methods regarding 1-min HRV recording windows. Prior research (78) has found that 10 s and 1 min rMSSD values correlate with 5 min rMSSD values. In general, HRV parameters that predominantly reflect parasympathetic cardiac modulation (rMSSD and pNN50) can be reliably measured using 1 min recordings; recordings of 1 min should be seen as the absolute minimum to obtain reliable hfHRV assessment (17, 79–81). Given that our primary HRV parameter of interest was rMSSD as it pertains to cardiac vagal function, utilizing 1-min recording windows was acceptable (17, 45, 82).

In addition to the apparent limitations of this trial, there are several important study strengths to be noted. Reducing clinical visitation time, HRV recording time, and daily treatment delivery time may have been a significant contributing factor to increasing treatment compliancy and achieving high clinical visitation attendance; MDB was self-delivered with a novel respiratory-gating device able to monitor the respiratory dynamics of each participant (35, 83–86). Noninvasive tVNS treatment was utilized to increase patient safety, usability, and decrease risk for adverse events (38). It has been proposed (87) that short- term meditative-based practices could have a more promising effect upon clinical outcomes as compared to continual long-term meditation practices (such as the 8-week mindfulness-based stress reduction program, MBSR) for specific vulnerable populations (88). tVNS treatment durations commonly range from 30 min to 2hours per session (89, 90). However, to match dosage and vagus nerve innervation across all groups, tVNS and MDB were practiced for 30 min per day split into two 15 min sessions; delivering the treatments in this manner may have helped increase treatment useability and compliancy but may have also changed the way in which the vagus nerve imparts clinical effects over time.

### Conclusions

Meditative-based diaphragmatic breathing and vagus nerve stimulation had no significant across-group effects on ultra short-term PPG-measured cardiac vagal tone among adults with FM. No significant across group differences were found for changes in average self-report NRS pain intensity; active MDB as well as sham tVNS resulted in the largest improvements in average pain intensity at 2-weeks when compared to sham MDB and active tVNS. The absence of significant across-group changes in ultra short-term PPG-measured cardiac vagal tone in response to vagal-innervation treatments in those with FM may indicate that more reliable long-term recordings procedures should be utilized and/or that longer (i.e., >2-weeks) vagus nerve innervation treatment methods should be delivered. However, finding significant across-group differences for overall FM severity and current pain intensity may indicate that meditative-based diaphragmatic breathing and vagus nerve stimulation provide differentiable yet complementary approaches to noninvasive FM pain management. Further research should be conducted on evaluating long-term noninvasive vagus nerve innervation methods for treating FM due to their useability, portability, and potential effectiveness.

## Data availability statement

In accordance with institutional guidelines, anonymized data is available upon request with a corresponding application describing the intent and justification(s) for investigative analysis.

## Ethics statement

The studies involving human participants were reviewed and approved by Regional Committees for Medical Research Ethics South East Norway. The patients/participants provided their written informed consent to participate in this study.

## Author contributions

The manuscript was drafted by CP. Study was conceptualized and designed by CP and HJ. Critical revision of the manuscript for important intellectual content was conducted by CP, AS, LR, and HJ. Blinded statistical analysis was performed by LD. This study was conducted as CP's Ph.D. Fellowship with the principal supervision of HJ. Co-supervision of AS and LR. Data acquisition, analysis, and interpretation of data was performed by all authors. All authors contributed to the article and approved the submitted version.

## Funding

Funding for a doctorate fellowship was received from the South-East Regional Health Authority, Norway (Project Number: 2017/766) to conduct and analyze data from this study. The funding source had no role in the design and conduct of the study; collection, management, analysis, and interpretation of the data; preparation, review, or approval of the manuscript; and decision to submit the manuscript for publication.

## Conflict of interest

The authors declare that the research was conducted in the absence of any commercial or financial relationships that could be construed as a potential conflict of interest.

## Publisher's note

All claims expressed in this article are solely those of the authors and do not necessarily represent those of their affiliated organizations, or those of the publisher, the editors and the reviewers. Any product that may be evaluated in this article, or claim that may be made by its manufacturer, is not guaranteed or endorsed by the publisher.

## Footnotes

^1^ https://www.fmaware.org/fibromyalgia-the-economic-burden/
